# Matched whole-genome sequencing of blood (10×) and five single sperm cells (1×) per individual in 53 men

**DOI:** 10.1038/s41597-026-06808-0

**Published:** 2026-02-10

**Authors:** Weiming Chen, Lei Yu, Ruidong Li, Hao Su, Zongyu Chen, Zhixu Zhang, Hui Zhang, Xiaolan Zhang, Yani Ding, Feifei Gou, Yu Lu, Ye Pan, Yong Zhang, Jun He, Chaojun Chen, Zongjian Tan, Zhenyu Jia, Jianguo Zhu

**Affiliations:** 1 Guizhou University, Medical College, Guiyang, 550025 China; 2 Department of Botany and Plant Sciences, University of California, Riverside, Riverside, CA 92521 USA; 3 Urology Department, Guizhou Provincial People’s Hospital, Guiyang, 550001 China; 4 Urology Department, Huaxi Area People’s Hospital, Guiyang, 550025 China; 5 Obstetrics Department, Huaxi Area People’s Hospital, Guiyang, 550025 China; 6 Gynecology Department, Guizhou Provincial People’s Hospital, Guiyang, 550001 China; 7 Obstetrics Department, Guizhou Provincial People’s Hospital, Guiyang, 550001 China; 8 Reproductive Department, Guizhou Provincial People’s Hospital, Guiyang, 550001 China

**Keywords:** Genome informatics, Experimental models of disease

## Abstract

Asthenozoospermia, characterized by reduced sperm motility, is a major contributor to male infertility and motivates improved resources for studying spermatogenesis at the genomic level. Here, we present a paired whole-genome sequencing (WGS) dataset from 53 Han Chinese men, comprising matched blood WGS per participant (target ~10×) and 3–5 low-coverage single-sperm WGS libraries per participant (target ~1×). The dataset includes 263 single-sperm libraries (79 from 16 asthenozoospermic participants and 184 from 37 normozoospermic participants) and is accompanied by rich participant-level metadata, including baseline characteristics, endocrine measurements, and semen parameters such as sperm motility and vitality. Raw reads underwent standardized quality-control filtering, and key sequencing metrics (Q20 and GC content) met commonly used thresholds; the achieved mean depth was approximately 10× for blood and ~1.7× for single sperm. By integrating sperm motility/vitality phenotypes with individual-matched genomic information, this resource provides a foundation for male reproductive genomics and for developing and benchmarking algorithms for gamete-genome dissection, and may support future translational research on male infertility evaluation.

## Introduction

Male infertility is a growing global health concern, affecting approximately half of all infertile couples^1^. This complex condition is often associated with impaired sperm production and quality, driven in part by genetic variations such as chromosomal abnormalities and single-gene mutations^2,3^. Genetic causes account for an estimated 10–15% of male factor infertility and are particularly prevalent in men with azoospermia and severe oligozoospermia^4^. However, many cases remain uncharacterized due to diagnostic limitations and incomplete understanding of the molecular underpinnings of spermatogenic failure^5^. Among semen abnormalities, asthenozoospermia, which is characterized by reduced sperm motility, is especially common, affecting over 20% of infertile men and accounting for 50.5% of cases in large clinical cohorts^6^. Teratozoospermia and low semen volume are also frequently observed, reported in 54.1% and 8.4% of infertile men, respectively^7^.

To advance our understanding of male reproductive genetics, single-cell whole-genome sequencing of sperm has emerged as a powerful tool^8^. Sperm’s haploid nature is particularly valuable, allowing precise analysis of genetic events without needing parental samples, which simplifies research and addresses ethical concerns^9–11^. This technology offers high accuracy in identifying genetic variations and can detect issues like sperm mosaicism and morphological variations^9,12,13^. While established trio-based methods are reliable for clinical diagnosis and *de novo* mutation detection^14,15^, they face challenges with cost and accessibility^16–18^. Similarly, the widespread clinical application of single-sperm sequencing is constrained by cost and the technical demands of high-resolution analysis, especially for complex variants^9,19^. Although low-coverage sequencing can reduce costs and offer advantages in patient privacy^16,18,20^, it presents its own challenges for comprehensive variant detection^21^.

To address these limitations and provide a robust resource for the development of single sperm sequencing methodologies, we developed a specialized paired genomic dataset. This dataset comprises 10× whole-genome sequences from blood samples and 1× single-cell whole-genome sequences from individual sperm cells obtained from 53 Han Chinese males, including 37 normozoospermia controls and 16 asthenozoospermia patients. Notably, all samples were confirmed SARS-CoV-2 free at the time of collection. This ensures that the clinical phenotypes, particularly sperm motility and concentration, represent a baseline state unaffected by the potential confounding effects of recent viral infection on male reproductive parameters. This paired genomic resource facilitates the development and benchmarking of algorithms for single-cell data imputation and physical haplotype phasing. By providing matched somatic-germline data, it serves as a specialized benchmark for investigating meiotic crossover patterns and validating the technical feasibility of low-pass single-sperm sequencing in reproductive genomics^22–26^.

## Methods

### Ethical approval

This study was approved by the Ethics Committee of Guizhou Provincial People’s Hospital (Approval Number: Research [2019]61). All procedures involving human participants adhered to the ethical standards of the 2013 Declaration of Helsinki and its later amendments. Informed consent was obtained from all 53 participants prior to sample collection. The cohort included 16 men diagnosed with asthenozoospermia and 37 fertile men as controls. Participants were recruited from the Reproductive Department (ART clinic) of Guizhou Provincial People’s Hospital (patients) and the local community (fertile controls). Prior to sample collection, written informed consent was obtained from all 53 participants, explicitly specifying their consent for both study participation and the public sharing of genomic data in international repositories. To protect participant privacy, samples were labeled using a de-identified coding system, and all records containing personal identifiers are managed independently by the hospital’s Ethics Committee, ensuring they remain inaccessible to the research team.

### Clinical data collection

#### Baseline data

Baseline characteristics for all participants included demographic information (age, height, weight, smoking status, drinking status) and, where applicable, reproductive history (gravidity, parity). Laboratory parameters collected comprised blood markers such as white blood cell count, red blood cell count, progesterone, estradiol, follicle-stimulating hormone (FSH), luteinizing hormone (LH), prolactin, and testosterone. Semen analysis parameters included semen volume, liquefaction time, abstinence period, sperm concentration, total motility, progressive motility, non-progressive motility, immotile sperm, and motile sperm count. Additionally, detailed sperm velocity and kinematic parameters were recorded: average path velocity (VAP), curvilinear velocity (VCL), straight line velocity (VSL), mean angular displacement (MAD), linearity (LIN), straightness (STR; VSL/VAP), wobble (WOB), amplitude of lateral head displacement (ALH), beat cross frequency (BCF), and rapid linear concentration. All participants were of Han Chinese ethnicity and reported no history of COVID-19 infection.

#### Inclusion and exclusion criteria

**Fig. 1:**
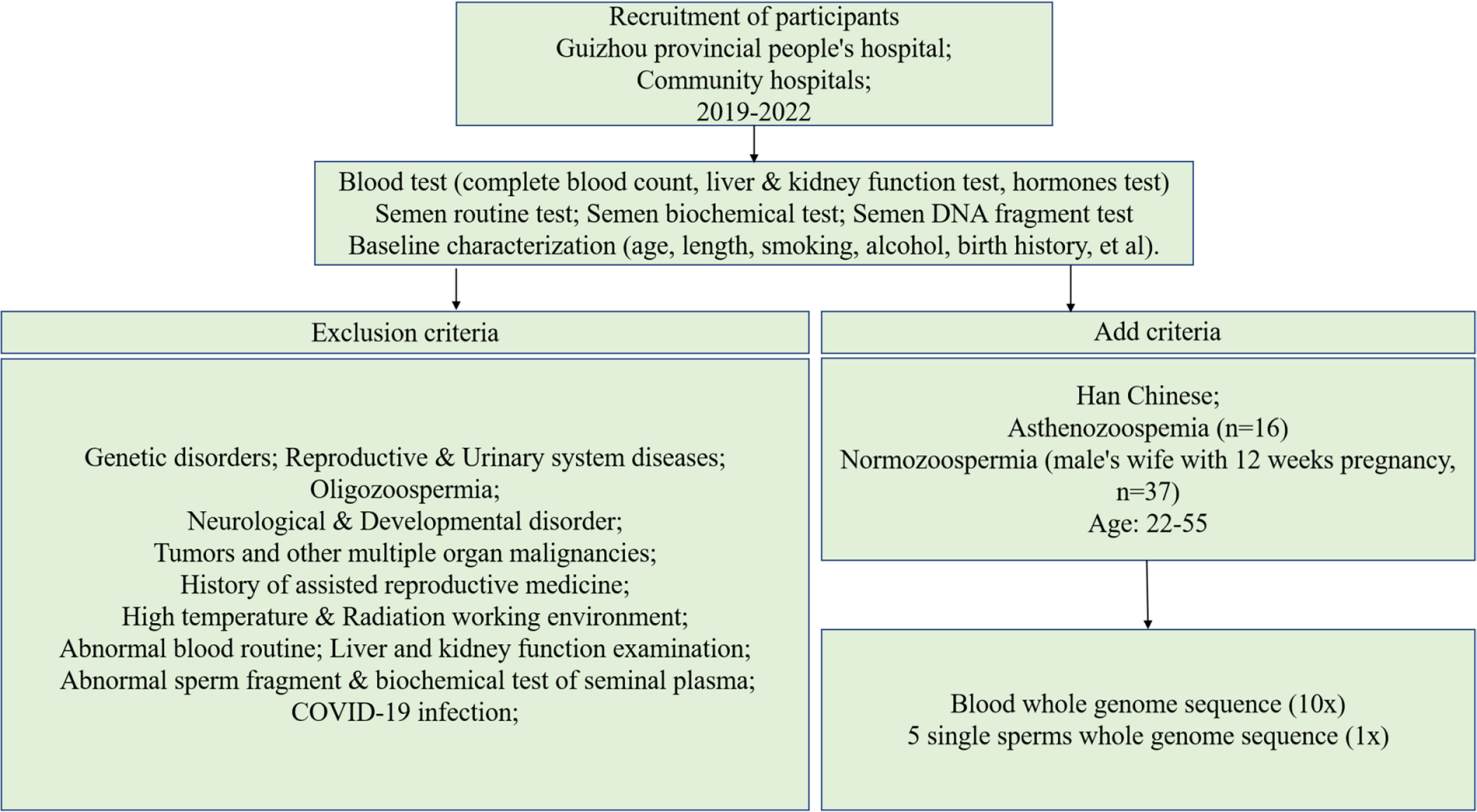
Participant Recruitment Workflow.

The inclusion and exclusion criteria for this study are detailed in Fig. 1. All participants were of Han Chinese ethnicity. We excluded individuals with a history of previous illnesses, abnormal blood tests, or seminal plasma abnormalities.

1. Asthenozoospermia Group: Participants in this group were diagnosed according to the World Health Organization (WHO) Laboratory Manual for the Examination and Processing of Human Semen (5th edition). Diagnostic criteria required an abstinence period of 2 to 7 days, with sperm motility assessed in three consecutive tests. Thresholds for diagnosis were total motility <42% or progressive motility <32%, alongside a sperm concentration >15 × 10^6^ sperms/mL.
2. Control Group: This group comprised men whose partners had a gestational age of 12 weeks or less, confirmed by positive blood HCG test results. Criteria for normozoospermia included progressive motility >32% or total motility >42%, with a sperm concentration >15 × 10^6^ sperms/mL.

#### Sample naming

We followed a standardized system for sample naming, as shown below.

1. Blood Samples: Names included the group (AS for asthenozoospermia, NF for normozoospermia), followed by age and a sequential sample ID. For example, “AS32-1” and “AS32-2” denote blood samples from two different asthenozoospermia participants aged 32. Control group samples were labeled sequentially (e.g., “NF1”, “NF2”, “NF3”).
2. Single Sperm Samples: Names included the group, age, and sperm number. For instance, “AS32-s1” to “AS32-s5” represented single sperm samples from an asthenozoospermia participant aged 32, while “NF28-s1” to “NF28-s5” were from a 28-year-old control participant. Sperm samples were consistently numbered 1–5, with specific DNA extraction details for each numbered sample available online^27^.

### DNA extraction and sequencing

#### Sperm isolation procedure

1. Semen Washing: After complete semen liquefaction (30 minutes), 10 μL of semen was pipetted into a PCR tube containing 50 μL of phosphate-buffered saline (PBS). The mixture was thoroughly mixed and centrifuged at 3000 rpm for 30 seconds.
2. Sperm Dilution: The supernatant was removed, leaving approximately 5–10 μL of sediment. The upper part of the sediment was carefully aspirated and transferred to a 1.5 mL Eppendorf tube. The sperm pellet was then diluted with PBS to a total volume of 1 mL.
3. Concentration Adjustment: Based on the sperm density determined by routine semen analysis, the sample was further diluted with PBS to achieve a concentration of 10–20 sperm per 100 μL. Microscopic observation was used to verify a concentration of approximately 1 sperm per 3–5 fields of view.
4. Preparation of Isolation Droplets: The diluted sperm suspension was thoroughly mixed. Using a micropipette, 100 μL of the suspension was drawn and deposited into uniform droplets, approximately 3 mm in diameter, onto a culture dish within 20 seconds. These droplets were then covered with paraffin oil to prevent evaporation.
5. Single Sperm Picking: A selection needle was primed in saline solution. Under an inverted microscope, each droplet was inspected. Upon identifying a droplet containing only a single sperm, the sperm was aspirated into the needle and transferred to a PCR tube. Post-picking checks were performed to confirm no sperm remained in the droplet or needle. For each male participant, 10 single sperm were isolated: 5 for immediate sequencing and 5 as −80 °C backups. During isolation, efforts were made to avoid sperm with visible morphological abnormalities, such as incomplete heads, empty heads, or degenerated sperm with blackened cytoplasm.
6. Blank Control: To ensure methodological rigor, blank control droplets (without sperm) were included. Any blank droplets subjected to picking were recorded as negative controls. The absence of sperm in these blanks was confirmed using agarose gel electrophoresis after library preparation.

#### DNA Quality control and sequencing

1. Single Sperm DNA Sequencing: For each individual, 5 single sperm were selected for library preparation using the PicoPLEX DNA-seq Kit. Single-sperm whole-genome sequencing was performed on the BGISEQ-2000 platform at approximately 1x coverage, generating paired-end 150 + 150 bp reads with 10 bp barcodes.
2. Blood DNA Extraction and Sequencing: Genomic DNA from blood samples was extracted using a standard protocol involving red blood cell lysis, centrifugation, and subsequent nuclear lysis with Proteinase K/SDS (1-hour incubation). After cooling, DNA was precipitated with phenol/chloroform, followed by cold isopropanol/NaOAc. The DNA pellet was then centrifuged, washed with 75% ethanol, briefly dried, and dissolved in TE buffer. Whole-genome sequencing (WGS) of blood DNA was performed on the BGISEQ-500 platform with 10x coverage, generating paired-end 150 + 150 bp reads with 10 bp barcodes, and following the DNBSEQ library construction protocol.
3. DNA Quality Control: DNA quality was assessed by measuring both concentration and integrity. DNA concentration was determined by spectrophotometric measurement of absorbance at 260 nm and 280 nm. DNA integrity was evaluated using 1% agarose gel electrophoresis, run at 150 V for 40 minutes. All sequencing and quality control procedures were conducted according to the service provider’s standard workflow. All data were sequenced to the predetermined coverage specifications without further sequencing.

#### Data quality control

Extracted DNA from both blood and sperm samples underwent quality control via microplate reader assay and gel electrophoresis, with all results meeting the criteria for library construction. Notably, single sperm samples showed evidence of protein contamination, necessitating purification with magnetic beads prior to library construction and sequencing. Of the 55 subjects initially recruited, 2 withdrew due to personal reasons, resulting in the successful sequencing of DNA from 53 blood samples and 263 sperm samples.

The DNA concentration and integrity metrics for all blood and sperm samples are provided in the technical validation files at figshare^27^. These records include microplate reader assay results, gel electrophoresis lane coordinates, and the corresponding full-length electrophoresis images used for quality verification.

## Data Records

The whole-genome sequencing datasets generated in this study have been deposited in the Genome Sequence Archive for Human (GSA-Human) under project accession number HRA005452^28^. A comprehensive metadata mapping file linking individual subject codes to their corresponding library (read sets) is available at figshare^27^.

## Technical Validation

To facilitate phenotypic characterization and genomic correlation studies by data scientists, we have provided baseline demographic and clinical data for all 53 participants. This table highlights the differences in hormone levels and sperm motility parameters between the Asthenozoospermia (AS) and Normozoospermia (NF) groups.

No significant differences were observed in hormone levels, including progesterone (P), estradiol (E2), follicle-stimulating hormone (FSH), luteinizing hormone (LH), prolactin (PRL), and testosterone (T) (*P* > 0.05). However, significant differences were identified in key sperm motility parameters. The NF group demonstrated significantly higher total motility (48.42 ± 22.46% vs. 20.82 ± 9.12%, *P* < 0.001), curvilinear velocity (VCL; 66.42 ± 17.69 µm/s vs. 56.59 ± 10.65 µm/s, *P* = 0.044), and mean angular displacement (MAD; 26.58 ± 17.23° vs. 9.90 ± 6.89°, *P* < 0.001) compared to the AS group. Other motility parameters, including VAP, VSL, LIN, STR, WOB, BCF, and ALH, showed no significant differences between groups (*P* > 0.05). These findings, summarized in Table 1, confirm superior sperm motility performance in the NF group with comparable hormone levels across both cohorts. Outcome descriptive continuous variables are presented as Mean ± SD, and categorical variables as N (%). To ensure transparency and facilitate future research, the raw fastq data used in this analysis have been made available^27^.

**Table 1:** Samples base motility characterizations.

Variable	Mean ± SD		*P*-value
	A group	N group	
n	16	37	—
P(nmol/l)	1.15 ± 2.56	0.39 ± 0.11	0.072779
E2(pmol/L)	50.59 ± 23.44	58.95 ± 21.16	0.207390
FSH(IU/L)	4.29 ± 1.79	3.81 ± 1.33	0.284559
LH(IU/L)	3.10 ± 1.30	3.27 ± 1.35	0.680196
PRL(μg/L)	12.75 ± 8.05	11.26 ± 4.60	0.398381
T (nmol/L)	17.19 ± 5.08	16.31 ± 5.59	0.591975
Total motility (%)	20.82 ± 9.12	48.42 ± 22.46	0.000018
VAP(μm)	31.39 ± 6.22	36.72 ± 12.35	0.108403
VCL(μm)	56.59 ± 10.65	66.42 ± 17.69	0.044447
VSL(μm)	26.48 ± 6.29	31.83 ± 12.10	0.101477
MAD (°)	9.90 ± 6.89	26.58 ± 17.23	0.000483
LIN (%)	42.61 ± 7.64	43.34 ± 10.81	0.807554
STR (%)	74.62 ± 9.98	76.18 ± 10.80	0.625337
WOB (%)	51.00 ± 8.13	52.13 ± 9.88	0.689486
BCF (times/s)	4.56 ± 0.91	4.57 ± 0.75	0.953746
ALH(μm)	1.82 ± 0.43	2.13 ± 0.66	0.100425

Note: AS group(asthenozoospermia); NF group (normozoospermia); Total motility (percentage of moving sperms in total sperm number); Forward moving (percentage of forward moving sperms in total number). T test results between groups. P(progesterone); E2(estradiol); FSH (follicle stimulating hormone); LH (luteinizing hormone); PRL (lactogen); T(testosterone); VAP (average path velocity); VCL (Curve velocity); VSL (linear velocity); MAD (Mean angular displacement); LIN (linearity); STR (Forwardness of sperm motility, VSL/VAP), WOB (Parameters of amplitude and direction of oscillation in advancing spermatozoa); flagellar beating frequency(BCF); ALH (lateral oscillation amplitude). Data are expressed as mean ± standard deviation (SD). A two-sample t-test was used, with *P*-values < 0.05 considered statistically significant. Significant differences are observed in Total motility (*P* < 0.001), VCL (p = 0.044), and MAD (*P* < 0.001) between the two groups.

Raw sequencing data underwent standardized quality control filtering, including contaminant and adapter removal, and elimination of low-quality reads, consistent with genomic studies of sperm samples. For blood data, the average Q20 (%) was 97.35 ± 0.37 for the AS group and 97.64 ± 0.81 for the NF group. Average GC content (%) was 40.44 ± 0.26 for AS and 40.52 ± 0.16 for NF. For sperm data, the average Q20 (%) was 96.24 ± 0.87 for the AS group and 96.76 ± 0.68 for the NF group, with average GC contents (%) of 39.66 ± 0.74 for AS and 39.48 ± 0.79 for NF. All these metrics met established quality requirements for downstream analysis^29^.

Clean bases from the filtered data were used to verify sequencing coverage. The actual average coverage achieved was 10.3x for blood samples and 1.7x for sperm samples, consistent with target coverage commonly employed in reproductive cell sequencing protocols. Specifically, asthenozoospermic (AS) and normozoospermic (NF) blood samples had average coverages of 10.75 ± 0.22 and 11.39 ± 0.50, respectively. Single sperm samples showed average coverages of 1.85 ± 0.25 for AS and 1.81 ± 0.23 for NF (Table 2).

**Table 2:** Blood and sperm Whole-genome sequence coverage.

Sample type	Group	Sample Size (N)	Coverage
Blood	AS	16	10.75 ± 0.22
	NF	37	11.39 ± 0.50
Sperm	AS	79	1.85 ± 0.25
	NF	184	1.81 ± 0.23

## Usage Notes

Access to this dataset (accession number HRA005452) is managed through the National Gene Bank of China. Before downloading, users are requested to read the official usage instructions and guidelines available at https://ngdc.cncb.ac.cn/gsa-human/document/GSA-Human_Request_Guide_for_Users_us.pdf. The dataset is directly available for download via the following repository link: https://ngdc.cncb.ac.cn/gsa-human/s/7OzjPJ1z. This direct link points to the dataset and includes complete information regarding the Data Access Committee (DAC). The dataset is released under a Creative Commons Attribution 4.0 International (CC BY 4.0) license.

## Data Availability

The whole-genome sequencing datasets generated and analyzed during the current study are available in the Genome Sequence Archive for Human (GSA-Human) at the China National Center for Bioinformation (CNCB)/Beijing Institute of Genomics (BIG), Chinese Academy of Sciences, under accession number HRA005452. The data can be directly accessed and downloaded via the following repository link: https://share.cncb.ac.cn/7OzjPJ1z/HRA005452/^28^. Additional metadata and technical validation records are available at figshare^27^.

## Code availability

The code used for quality control and data preprocessing is available^27^.
